# Emerging work environments in the pandemic era: a gendered approach to work-life balance programs

**DOI:** 10.3389/fsoc.2023.1120288

**Published:** 2023-04-18

**Authors:** B. Sreya, Ayyagari Lakshmana Rao, G. Ramakrishnan, Nikhil Kulshretha

**Affiliations:** ^1^SRM University, Amaravathi, India; ^2^Doon Business School, Dehradun, India

**Keywords:** working from home, work-life balance, psychometric testing, satisfaction level of employees, COVID-19 pandemic

## Abstract

As the coronavirus pandemic affects virtually every sector of the economy, this ongoing review examines the effects of remote working on women's job performance—including hypotheses about serious activities and how they may balance work and family. In recent years, psychometric testing has become increasingly popular with organizations worldwide, and they are looking at this method to better understand how women achieve balance in their lives. The aim of this work is to investigate how different aspects of psychometrics and factors relating to work-life balance influence women's satisfaction levels. An exploratory factor assessment (EFA) and a confirmatory factor assessment (CFA) using a seven-point Likert scale were performed on data collected from 385 selected female IT workers whose satisfaction levels toward psychometric assessments in their organization were examined. The current study uses EFAs and CFAs to develop and identify the key factors in women's work-life balance. The results also showed that three significant variables accounted for 74% of the variance: 26% from work and family, 24% from personal factors, and 24% from loving their job.

## 1. Introduction

Assessing an employee's willpower is often novel and has several diverse connotations (e.g., Saks, 2006; Shuck, 2006). In research, three types of employee willpower have been identified. Employees' willpower (amicable enthusiasm) is influenced by a number of variables, including hierarchical responsibility, perspective affiliation, and workaholism. Despite the prominence of these trends, discussions have emerged around what should serve as a representation of employee willpower or the estimated amount of it (Hallberg and Schaufeli, 2006; Macey and Schneider, 2008; Saks and Gruman, 2021). In model one, Kahn and Fridkin (1996) conducted a subjective examination of balancing staff members and advisors in an employee structure to discern how personnel differs in terms of their interests and perspectives. Kahn identified the “favored self” in everyday activities in accordance with Goffman's (1961) viewpoints, techniques, connections, and separations. The “favored self” highlights the well-known personality and behavior individuals decide to embody in a variety of jobs. As the study progressed, Kahn noticed employees expressing high intellectual capabilities within their job duties. He also characterized employees' willpower under three attributes that personnel managers value and use themselves. According to a model by Maslach et al. (1997), burnout is the opposite of willpower, and willpower has three attributes: energy, inclusion, and viability. These are also the opposites of three elements of burnout: weariness, negativity, and absence of viability. In this manner, they cautioned that willpower and burnout might be predicted by using the Maslach Burnout Stock (Maslach et al., 1997). Immediately, Schaufeli et al. (2002) reclassified worker willpower using the attributes force, devotion, and retention, by developing the Utrecht Work Commitment Scale (UWES) to determine willpower and burnout. In contrast to MBI's lack of viability aspect, commitment implies having a sense of obligation, excitement, and importance in one's perspective. Retention alludes to strong realization and development of perspective about filing in within an unspecified amount of time. However, previous studies have extended the assembly to incorporate perspectives and hierarchical willpower. Saks (2006) characterized employees' willpower as a change and super form that contains highbrow, profound, and behavioral attributes related to the hobby execution of a character. Rothbard (2001) and May et al. (2004) advocate that humans have exceptional jobs in establishments and hierarchical elements of hobbies. Following this, Saks (2006) defined hierarchical willpower as a sense of a personal connection to the organization in the context of the character's talented jobs of the character withinside the union. As stated in the alternate social hypothesis (SET) (Cropanzano and Mitchell, 2005), under situations of collaboration, individuals exhibit character and affiliation through gathering trust and sharing duties. Like this, the benefits and assets derived from the association result in a corresponding connection among people and collaboration, even though in doubt, of progress. Because people perceive that they must give back to the organization in kind to get a bonus from the manager or company (such as support, equity, and acknowledgment), there is an increase in group willpower Saks, 2006; Saks et al., 2022). Macey and Schneider (2008) considered authoritative willpower as a highbrow state and characterized it as an improvement of perspectives on willpower, achievement, portray obligation, conventional obligation, portray achievement, and educational strengthening. According to Thomas (2009), willpower surpasses an employee's achievement or commitment; it highlights the character's delight and hierarchical citizenship behavior as an improved mode of thinking and acting (Pitt-Catsouphes and Matz-Costa, 2008; Thomas, 2017).

Psychometric tests measure a candidate's intellectual ability, character, or behavior and determine whether they are capable of achieving a certain goal or quality. Psychometric tests rely on specific structures (e.g., mathematical, mechanical, sensory, or verbal). Psychometric exams are usually offered online, but they may also be administered face-to-face at an assessment center. Assessments are made to simplify the recruitment process and efficiently identify top candidates. Currently, they are a common obstacle, especially when applying for graduate programs. These examinations are often administered in pairs, with the selection being chosen based on the approach of the professional setting to which the candidate is applying. Psychometric scores are often considered alongside candidates' resumes, cover letters, and unique evaluation adjustments, such as more specialized, location-specific projects, simulated conditions, and in-depth interviews. The benefit of performing well on psychometric testing should not be ignored. A very high psychometric rating dramatically increases the chances of getting a final interview. Psychometric evaluations objectively and quantitatively assess an individual's traits, abilities, capabilities, behavior, style, and inclinations. They are widely used in professional publications and perspectives to determine a person's abilities and find a more favorable profession or job. It is critical to ensure legitimacy, reliability, and norms; and these should be included in any psychometric test. As organizations are becoming more selective about who they hire, psychometric evaluations have become a regular component of the recruitment process. This is to ascertain the suitability of potential employees. Additionally, psychometric assessments can influence any part of an employee's life cycle, beginning with recruitment, preparation, and follow-up.

### 1.1. Objectives of the study

- This study aims to explore the factors of WLB and to seek the importance of the psychometric test in current situations.
- To identify the impact of working from home on the satisfaction level of female employees.
- To find out the relationship between the adoption of psychometric tests and the satisfaction level of employees.

### 1.2. Hypothesis of the study

- **H0**_**1**_ There is no effect of working from home on the satisfaction level of female employees.
- **H0**_**2**_ There is no relationship between the adoption of psychometric tests and employee satisfaction.
- **H0**_**3**_ There is no difference in the opinion of the satisfaction of the female employees in the selected samples based on their age.

### 1.3. Determination of sample size

In Table 1, the study's sample size is determined as follows: approximately 50,000 women are working in the IT sector at Infopark, Technopark, and Kinfra Park. A total of 499 questionnaires were distributed and the response rate was 77, 15%, or 385.

**Table 1:** Sample.

**No**	**Indian companies from Kerala**	**Questionnaires distributed**	**Valid questionnaires**
1	Infopark	187	123
2	Technopark	167	142
3	Kinfra Park	145	120
Total		499	385

Response rate: 77.15%.

Primary data analysis.

Source: Computed from primary data.

## 2. History of psychometric assessments

Conclusions about the value of psychometric tests can be inferred from human reports spanning social groups, geographies, and faiths. Three eminent specialists, Alfred Binet, Victor Henri, and Theodore Simon developed in France the state-of-the-art psychometric test that is used today throughout the 19th century. Following this, military forces started to use them everywhere in Western countries. The Woodward (1917) was created as a result of officials choosing the most plausible candidates. Subsequently, the “Big Five” test was devised in late 1950, and it has since become one of the most well-known trait models. The future of Psychometrics includes aspects of surveys intended to build importance and responsibility.

### 2.1. Adaptability and effectiveness of psychometric tests

A psychometric test assesses a person's cognitive abilities and personality traits, helping to determine whether they possess the traits and skills necessary to be successful as a business visionary, who can benefit from recent advancements and serve as open-door experts in the public sphere (Ratten, 2020). Psychometric testing can help organizations understand aspects of intellectual ability and social style that discussions and meetings cannot. A psychometric test is a standard of logic and strategy used in both academic and clinical settings. It also offers an unbiased assessment of a variety of limitations, including Smart Thinking, Clear Industry Fit, Clear Job Traits, Character Type, and others. Although psychometric tests date back to ancient times, the current psychometric test was developed by analyst and clinician Francis Galton. Galton, also called “the father of psychometrics”, was among the first to use this specific term. He created a method to assess a person's motor and tactile abilities. Galton's work was further developed by James McKeen Cattell, who referred to psychometric tests as “mental tests”. Current psychometric testing began in France in the 19th century, when clinicians used them to separate patients with mental disorders. Alfred Binet, Victor Henri, and Theodore Simon, three French doctors, conducted pioneering work in the field of psychometrics by developing a test to study cognitive and verbal abilities in children with mental illness. Over the course of their 15-year study, they made numerous breakthroughs that shed light on various aspects of human character. This test, known as the “mental hurdle test,” later became the basis for the Binet-Simon test, which was renamed the “Stanford-Binet Intelligence Scales” by Stanford psychologist Lewis M. Terman in 1916. The latest version of this long-established psychometric placement test was released in 2003 and is still widely used for admissions and selection purposes.

### 2.2. Process

We conducted a descriptive mechanism study with 385 samples. All selected respondents were women, and we administered appropriate psychometric evaluation questionnaires. A total of 499 questionnaires were distributed to obtain 385 valid samples.

## 3. The IT sector and COVID-19

In recent years, technology has had a significant impact on social orders' functional and merciless climate for a long time. For the purposes of this review, IT abilities are defined as utilizing IT tools and data hotspots to analyze, manage, introduce, and display data, in addition, to estimating and controlling outside events (Benzie, 1997). Moreover, (Vaishya et al., 2020) discussed using artificial reasoning for COVID-19 on the board. The impact of IT on medical care and the high commitment of innovation organizations are referenced in Javaid et al. (2020). Based on their research on accessible writing, Padikkapparambil et al. (2020) inferred that the Internet of Things (IoT) proved helpful for locating side effects and rapidly giving treatment. The novel Coronavirus disease caused technological changes in mid-2020 and fundamentally altered global society (Albahri et al., 2020). The new standard suggested to the general public that physical and social isolation was important in helping to stop the spread of the disease, while the worldwide economy should remain unchanged. Massive changes in media communications, technological innovations, and the emergence of new IT organizations were predictable and reflected how employees connect and act in the public arena. Business experts are the beneficiaries of many of these advances as they work as open-door experts in the public sphere (Ratten, 2020). They did not benefit from changes like those brought on by the COVID health pandemic, but they had the power to significantly alter the way business was being conducted. The effects of the virus have had both social and financial repercussions. Many ill-equipped organizations in the IT sector have crumbled under the new required working conditions. Before the situation, the most important IT organizations in India and the United States expanded in the direction of digitization when COVID's mode of transmission came to light (Ting et al., 2020). Workers who are currently far away due to the emergency are more vulnerable to the coronavirus, and close-by charge suggestions to ascend for laborers (Ashraf, 2020). Also, charge consistence activities should slack essentially, as recently far away staff needs very much planned to get passage to data, especially in expanding global areas. Estimates and trade executions by financial organizations will be significantly impacted in the near future by changes to profit articulations that take into account short-term misfortunes. Also, Siddiqui and Siddiqui (2020) defined financial incorporation as “the way of ensuring get passage to monetary contributions and very much planned and sufficient FICO assessment in which needed through the method for susceptible organizations which incorporates more fragile segments and low-profit organizations at a low evaluated cost” **[sic]**, which include reserve funds, FICO credit scores, settlement, protection, and financial competence, in addition, having an economic organization account. Due to their financial vulnerability, the majority of people in the world know that having their own money is their most reliable indicator of social wellbeing. Families living in poverty struggle to access financial assistance. Finance is a machine relating to public utilities beneath low rivalry cases with relatively few providers (Milana, 2012). Subsectors of age will present at this point and not be unaffected at some stage in this period. For the most part, the product program became influenced substantially less without a moment's delay than different subsectors because it now again appreciates conveying chain inconveniences that different troubled subsectors, which incorporates portfolio control in IT acquirement. A few projects will appreciate because the current situation will increment reliance on age. Under those circumstances, portfolio decision control is fundamentally based on vulnerability files; business control has quickly changed. The virtual innovations used during the COVID-19 pandemic by companies to complete virtual tasks are regarded as that company's virtual abilities (de Oliveira Valério et al., 2020). A company's transition to a virtual environment includes two or three virtual abilities, the utilization of cloud computing for cost creation, relying on information-driven navigation through testing, shifting the point of convergence from product-based to transporter-based, and exploring different avenues for result-based business models site (Iansiti and Lakhani, 2017). A carefully changed association's enormous elements comprise individuals, undertakings, things, information, and the cloud (Blaschke et al., 2017). During the subsequent unfolding of the pandemic, IT businesses should be coordinated for the inordinate systems administration data transmission. Over the whole period, the media communications undertaking does massively effectively because the call for aiding contributions increments essentially (Ramelli and Wagner, 2020). On the other hand, the logistics technology undertaking faces postponement in shipments of advanced things and movement oversee guidelines. Numerous little age organizations must forestall ventures (Hanif et al., 2020). Furthermore, the pandemic is expected to have an enormous and long-lasting effect on biometrics companies, related age manufacturers, and markets (Carlaw, 2020).

## 4. Theories describing work-life balance

### 4.1. Spillover theory

For the past 20 years, the majority of perspectives on the balance between hypotheses have focused on good and bad overflow (Zedeck, 1992). Initially proposed by Wilensky (1960), the overflow model is related to the concept that there can be an “extension” of encounters from the circle of work to non-work perspectives such that the view of society enjoy the strategies of work and non-work perspectives for someone is certainly limitation less (Parker, 1971). “Positive Spillover” and “Negative Spillover” have been used to characterize overflow (Dijkstra et al., 2010). Positive spillover appears in writing under unique names such as expansion, hypothesis, commonality, personality, isomorphism, continuation, and coincidence (Staines, 1980). Positive overflow refers to significant events that result in pride and success in one area after occurring in another (Kumar and Janakiram, 2017). Impact characterizes the amount of overflow that one region of the way of life has on the adjoining area, e.g., the effect that perspectives. “Vertical overflow” has been used to refer to a current tool that alludes to the severe leveling down of association of areas of existence like perspectives, own unique family, pastimes, etc. Fulfillment or disappointment in a subordinate area gushes right into a superordinate room. An extensive presence being the most superordinate of the regions appears to be impacted the most (Sirgy et al., 2001). A few scientists have worked to calculate the overflow impact. Small and Riley (1990), who created the Work Spillover Scale (WSS), noticed the initial experimental percentage of balance between professional and amateur sports activities. Grzywacz and Marks (2000), perceiving every powerful and horrible overflow affects, fostered a 16-issue scale estimating the outcomes of the perspectives -personal own family overflow. It is modified into trailed through the manner of Kinnunen et al. (2006) four-issue model estimating horrible perspectives to personal own family overflow, unique horrible own family to pics overflow, realistic illustrations to the confidential manner of household overflow and personal influential own family to perspectives overflow. Several measures and scales have been developed to assess work-family conflict and its impact, including Greenhaus and Beutell's (1985) interdomain conflict model, which examines the appraisal of positive and negative spillover, and Higgins, Duxbury and Irving (1992) application of self-discrepancy theory to develop measures for positive, negative, and neutral work-family outcomes. While some initial studies have examined work-family conflict, The Guest (2002) argues that further research is needed to explore the causes, mechanisms, and consequences of work-family conflict using a systematic approach.

### 4.2. Conflict theory

Conflict theory, first developed by Karl Marx several decades prior to Greenhaus and Beutell, is based in part on the theory proposed by the latter in 1985 that satisfaction and accomplishment in one area of life lead to compensation from a broader perspective. This compensation is based on the knowledge that the two areas, lifestyles, and points of view are fundamentally opposed to one another and that they have different needs. Kahn et al. (1964) and Katz and Kahn (1978) describe views-style warfare as a type of integral warfare in which tensions from various viewpoints and family ties appear in the conflict in some ways. Utilizing the appropriate level of support at each of these levels will be more challenging than participating in a single task. Lastly, conjecture relates to scarcity because if there is a limited amount of large funding, it will probably be available to those who are assigned to specific jobs. Greenhaus and Beutell (1985) make a distinction between three types of war for their account of rivalry speculation: Time primarily based on total action; Stress primarily based on total fighting; and Behavior primarily based on total war. Time-sensitive conflicts arise with limited time, making it difficult to efficiently meet the demands of multiple jobs. Views and styles are affected by time-consuming factors such as long working hours, irregular shift schedules, and the difficulty of adjusting to schedules. Action-based stress results from intellectual hobbies, collaboration fatigue, and career burnout. A conduct-based fight arises when views are expressed with demonstrated ways of behaving in opposition to a family's task and conflict may arise between the two tasks (Roy, 2016). Research has also shown that the encouraging of antagonism between views and lifestyles is bidirectional. In other words, the gap between labor and views narrows gradually over time, causing a disruption in the process (Gutek et al., 1991). Conflicts negatively affect vision and the circle of relatives (Adams et al., 1996). Numerous studies have disproved the ramifications of the views-lifestyle conflict, including persistent frailty (Frone et al., 1997), depression and hypertension (Haynes and Marques, 1984; Thomas and Ganster, 1995), male-associated real issues (Burley, 1995), anxiety and irritability (Hertz, 1986) among others.

### 4.3. Border and boundary theory

Boundary theory stresses how individuals create, protect and modify limits in order to rearrange and symbolize the arena experience (Ashforth et al., 2000). Boundary theory is derived from Nippert-Eng (1996) humanistic perspective, in which he portrays how people can discover and assign meanings to different points of view while domesticating them and allowing them to coexist. Boundary theory implies that there are mental, bodily, and behavioral limits between the views and non-views elements of a person's existence, which body the two areas as being precise and distinct from one another (Allen et al., 2014). Combining these premises together with admiration for restriction speculation, Clark, 2000 presented her idea of labor/existence line speculation, in which human beings set up views and non-views in a way so that an equilibrium is likely to be reached between them. The notion here is based on the idea that “views” and “non-views” are separate, but that they impact one another. Boundary theory sees the two as connected along a continuum that extends from department to reconciliation, to a point where at the shaft of the department, the two regions are mostly disconnected, and at the apex of coordination, the two are probably viewed as indistinguishable (Voydanoff, 2005). To determine whether the boundaries between the two regions are porous and flexible we examine the wise judgment of the department and the benefits of membership. Adaptability refers to the ability of strains to transfer from one area to another, whereas penetration refers to how far the limits can allow for psychical or behavioral additives to pass from one room to another (Saarenpää, 2016). Boundaries can be adjusted through techniques like strategic scheduling, role sharing, seasonal views, and working from home (Cowan and Hoffman, 2007). Following his depiction of boundaries as penetrable and adaptive, Clark (2000) claimed that boundaries may be viewed as a continuum from beings (and stubborn) to powerless (adaptable and mixed), advocating that human beings may be portrayed as “line crossers” and “boundary managers”. Traditionally, people are viewed as line-crossers who have their areas of Work and circle of relatives. Border crossers are classified as Central Border Crossers and Peripheral Border Crossers. Focused line crossers are notably dominant in each area and may become effectively subsidiary to the focal people in each area. This unit is one of the dispersed investigations within the Boundary/Border speculation stream, where focal boundary crossers achieve the most outstanding views than perimeter line crossers (Donald and Linington, 2008).

### 4.4. Enrichment theory

Conflict-centered perspectives have heavily influenced work-life balance research in many of its case studies. Still, there was a significant change in the modern perspective as scientists began researching the predicted harmonious connection between views and existence. (Powell and Greenhaus, 2006) developed the improvement hypothesis to examine how enhancement strategies connect an individual's self-image with their family and their family's values, with each process leading to individual development, or it can be described as the progression of intellectual property over different stages of a cycle (Baltes et al., 2009). The analysis demonstrates each circle of development of views-to-own and relatives development. Some comparative actions, such as assistance and quality overflow, are reciprocal, but there may be an essential distinction. Advancement involves the acquisition of qualities and experiences that individuals can use when confronting problems of existence. Consequently, developmental speculation suggests that upgrading the process of performing in a single area depends on obtaining property in every other area. Furthermore, high-quality overflow depicts transactions of encounters, skills, states of thoughts, and methods of behaving that begin with one area and then onto the next. In both views, moving objects in overflow might not be sufficient to show the existence and enhance the presentation of the character in every other area (Carlson et al., 2006). Carlson et al. (2006) argue that while moving objects in overflow may not be sufficient to demonstrate the existence and enhance the presentation of character in every other area, assistance has a widespread impact, and incorporating components in a single region can produce gains that improve the performance of every other area. It is also possible to improve facilities for individual pleasure while receiving assistance. Powell and Greenhaus (2006) attest that development can manifest in one of the following paths: Affective and Instrumental. In the course of workers' Work, positive ways of behaving and emotions are exchanged among the group and within their circle. Instrumental views-existence development occurs whilst skills and methods of behavior acquired in a single area enhance the display and viability of that character in every other vicinity.

### 4.5. Facilitation theory

In facilitation theory, items related to one activity enhance or facilitate participation in a different activity (Voydanoff, 2004). Frone (2003) portrays it as a way to ease encounters, improve skills, and open doors to make cooperation more sincere in any job. Assistance is fundamental to the notion that a given task becomes more accessible with every additional load. Despite the reality that assistance is imagined as a hypothetical associate to look at the existence of war, this is no longer considered as inverse shafts in the hypothetical continuum (van Steenbergen et al., 2014). This speculation is rooted in the evaluation led by Barnett (1998), who looked at the possibility of assisting in describing a view-existence fit. Accordingly, Grzywacz (2002) suggests that help occurs when people and social frameworks employ a given approach to perform more (Grzywacz and Butler, 2005). According to Wayne et al. (2007), dedication, gains, and progressed concept are the three components that individuals invest in when committing to separate exercise areas. These components include developmental gains, complete feeling gains, capital increases, and effectiveness gains. Improvement in operation is defined as the enhancement of skills necessary for performance, such as critical thinking. Positive energy and an understanding of how one's unique operation in one area can benefit all other areas can lead to improved performance. Hammer et al. (2009) further note that personal and capital additions also occur as a means of providing help.

### 4.6. Segmentation and integration

Department-aggregate range speculation has its roots in Nippert-Eng's “departmental inclinations” (1996), where extreme jobs become members of extreme job departments. The departmental version holds that views and non-views do not affect each other, and the two areas are distinct (Guest, 2002). Piotrkowski (1979) argues that beliefs and lifestyles are separated by suppressing the associated mental states, behavioral patterns, and propensities within the living area and, in a similar way, by recommending specific behaviors, feelings, concerns, or joys within the living area. As a result, the division is a significant division of labor and lifestyle. In its previous structure, the department was visible alongside the regular/real locus. Still, cutting-edge research has proven that the department is a functioning, psychosocial system that separates the two universes (Roy, 2016). Division and incorporation were conceptualized as shafts on a continuum of stability between entertaining and severe activities (Ashforth et al., 2000). Combination theory suggests that distinct boundaries between opinions can create representations that relate to a higher level of everyday lifestyles, thoughts, and nearby lifestyles (Clark, 2000). Morris and Madsen (2007) aimed to examine more functional additives such as locality into the aggregate speculation, stating that “joining requires cutting-edge perceptions that reengineer traditional views—lifestyles, fashions and integrating all collaborators viz employees, laborers, households, and networks as dynamic participants.” **[sic]** Zerubavel (1991) identified both segmenters and integrators and described segmenters as people who seem to separate themselves by creating a mental wall. These people preserve their views and domestic life at home. As a result, he defined integrators as individuals who coordinate the additives of the two areas while removing any stumbling blocks. Ashforth et al., in addition to non-views, (2000) noted that peculiarities occur freely, but views are likely to be coordinated with non-views. In the same way, Nippert-Eng (1996) identified excessive task aggregates and task departments. Although a high level of tasks may suggest a nation, there may not be any distinction between what is domestic and what is part of the public sphere (Barnes-Mauthe et al., 2013). A large task department exists while the two areas are considered and dealt with as separate entities. Almost any task can fall upon the coordination department continuum, with excessive task departments and excessive task aggregates as limits, and also has a part of the exam devoted to the discipline of segmentation-integration theory.

## 5. Reliability analysis

Here, Tables 2, 3, show the reliability test of the constructs used in the questionnaire is analyzed; it is found that all the constructs have a good value, so the items are reliable for further analysis.

**Table 2:** Case processing summary.

		***N***	**%**
Cases	Valid	385	100.0
	Excluded	0	0.0
	Total	385	100.0

^a^List-wise deletion based on all variables in the procedure.

**Table 3:** Reliability statistics.

**Cronbach's alpha**	***N*** **of items**
0.696	78

### 5.1. Hypothesis I

***H0 There is no effect of working from home on the satisfaction level of female employees.***

Here, Tables 4, 5 show the satisfaction level of female employees is compared with the adoption of the work-from-home situation; it is observed that the ANOVA is significant, so the regression is taken into account. Conversely, the adoption of WFH increases employee satisfaction by 33%, which is a positive sign. Since the significance of the test is accepted, the null hypothesis is not considered.

**Table 4:** ANOVA^a^.

**Model**		**Sum of squares**	**df**	**Mean square**	***F***	**Sig**.
1	Regression	54.223	1	54.223	196.305	0.000^b^
	Residual	105.792	383	0.276		
	Total	160.016	384			

a Dependent Variable: WFH.

b Predictors: (Constant), satisfaction.

**Table 5:** Model summary.

**Model**	**R**	**R square**	**Adjusted R square**	**Std. error of the estimate**
1	0.582^a^	0.339	0.337	0.52557

a Predictors: (Constant), satisfaction.

### 5.2. Hypothesis II

***H0 There is no relationship between the adoption of psychometric tests and employee satisfaction.***

Here, Table 6 shows the level of satisfaction is compared with the adoption of the psychometric test among IT employees. The result of the correlation seems to be significant at the level of 0.000 and the value of the correlation is positive but not particularly high. The Pearson correlation of 0.385 does not seem to be very problematic since it is positive.

**Table 6:** Correlations.

		**Satisfaction**	**Psychometric_ test**
Satisfaction	Pearson correlation	1	0.385^**^
	Sig. (2-tailed)		0.000
	N	385	385
Psychometric_test	Pearson correlation	0.385^**^	1
	Sig. (2-tailed)	0.000	
	N	385	385

** Correlation is significant at the 0.01 level (2-tailed).

### 5.3. Hypothesis III

***H0***_**3**_ ***There is no difference in the opinion of the satisfaction of the female employees in the selected samples based on their age.***

Here, Table 7 shows the analysis found no difference in the opinion of female employees on satisfaction levels in the psychometrically applied IT firms. This is because the significance value is greater than the limit of the other assumptions considered.

**Table 7:** ANOVA.

	**Sum of squares**	**df**	**Mean square**	***F***	**Sig**.
**Satisfaction**					
Between groups	3.054	5	0.611	1.471	0.198
Within groups	157.372	379	0.415		
Total	160.426	384			

## 6. Factor analysis

The R-element method was applied to look at the association behind the increase in consultant engagement. The simple suspicions essential to the variable analysis have been organized into actual and genuine worries. Reasonable doubts are associated with some hidden shape within a given arrangement of things. Using the grounded speculation philosophy, the analysts were able to formulate designs within the factors suitable for further investigation with EFA. According to an essential analysis, Kaiser-Meyer-Olkin goodness of fit is 0.841 (Tabachnick et al., 2007; Hair et al., 2013), and the goodness of fit for Bartlett's sphericity test (x^2^ = 2,201.362, df = 42) is pivotal (^*^0.05), which allows for the use of variable tests in the study. Using the Varimax rotation, the head component exam focused on the element scale with a critical focus on information reduction. Models for determining the number of variables to be removed were derived from the inert root model, stage of distinction, and the screen plot test. To determine the factor adequacy, the KMO test is performed, and the KMO value is 0.841, which is deemed sufficient. The chi-square value also shows a significant acceptance value of 0.001. As a result, the factors used here measure the correct concepts.

The commonalities Table 8 in this study indicates that all statements have a reasonable fit, as it has a positive extraction value, which allows all statements to be retained.

**Table 8:** Factor loadings.

**Factors**	**Factor loadings**
My work does not interfere with my family's activities	0.723
I do not feel materially tired when I come home from work	0.758
My efforts do not affect the amount of time spent on my personal	0.691
I have adequate time to interact with family members due to work overload	0.744
I have time for my activities	0.693
I do not compromise on social activities	0.725
I have regular contact with my relatives and friends.	0.754
I never miss out on any weekend shopping or outings with my family	0.736
I enjoy my work	0.742
The workplace is good and friendly	0.821
I enjoy working in my company	0.785
Work makes me creative	0.712

Primary data analysis.

As shown here, the commonalities Table 9 identifies the intensity of the statements related to the factors and also provides a picture of how many variables contribute to the overall work-life balance in the factor loadings. Together, the first factor explains 26%, the second factor explains 24%, and the third factor explains 24%, for a total of 74%.

**Table 9:** Rotated component matrices.

**Statements**	**Factors**	**Extractions**
Work and family	My work does not interfere with my family's activities	0.824
	I do not feel materially tired when I come home from work	0.842
	My efforts do not affect the amount of time spent on my personal	0.822
	I have adequate time to interact with family members due to work overload	0.796
Personal life	I have time for my activities	0.782
	I do not compromise on social activities	0.811
	I have regular contact with my relatives and friends.	0.789
	I never miss out on any weekend shopping or outings with my family	0.841
Workplace enjoyment	I enjoy my work	0.841
	The workplace is good and friendly	0.725
	I enjoy working in my company	0.753
	Work makes me creative	0.741

Total variances elucidated: 74% variance.

Primary data analysis.

**Figure 1:**
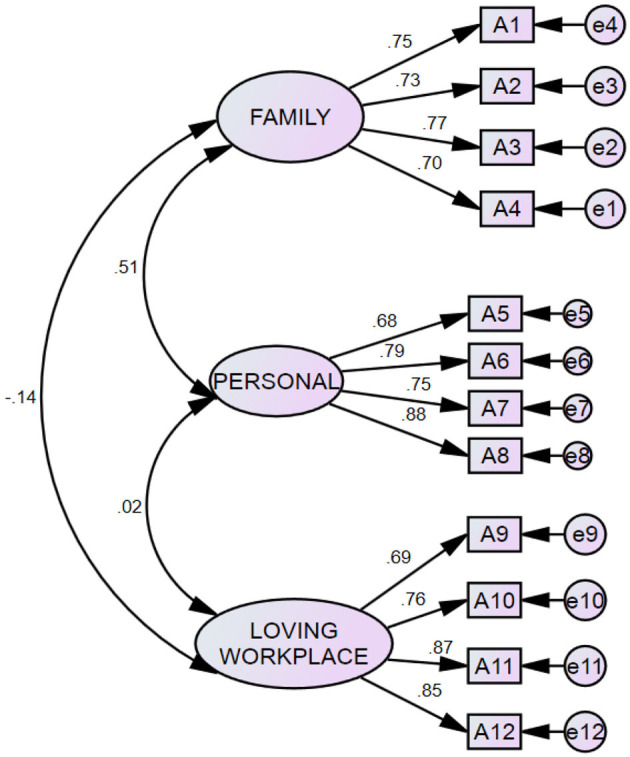
CFA of work-life balance.

In Figure 1, the researchers attempted the 3-issue version of the CFA. In the grounded hypothesis technique, naming the factors became difficult because elements were stacked on top of each other. There are three factors—arrangement, extent, and interest level—which include both employee commitment and elements separately. To facilitate a modern estimating model, the experts developed a generic model, which became prevalent, and required every idle variable to have four or more pointers (things). Direction estimations and their statistical significance, in addition to health indices, were used to assess the validity and health of the dimension version. Path estimates hyperlink the constructs to indicator variables, and the standardized loading estimates have to be at least 0.5 to verify that the signs are strongly associated with their related constructs and are one indication of assembling validity (Hair et al., 2013). The researchers used the standardized, most likely estimate to interpret the results.

Table 10 shows the model fit indices of the CFA measurements of the identified factors of work-life balance; these fitness references tell that the *P*-value is 0.702, which is within the acceptable range, and the CMIN value corresponding to DF is 0.886, also in the limit. The AGFI is 0.809, which shows a significant amount, and the CFI is 1.00. Finally, the RMSEA is significant at a 0.000% level; thus technically, this model is a fit.

**Table 10:** Fitness indices.

**CMIN/DF**	**AGFI**	**CFI**	**RMSEA**
0.886	0.809	1.000	0.000

Table 11 shows the values of the factor loadings of the statements in the CFA model; the values are presented as follows. My work does not interfere with my family's activities 0.75, I do not feel materially tired when I come home from work. 73, My efforts do not affect the amount of time spent on my personal needs. 77 I have adequate time to interact with my family members after my workload. 70, I have time for my activities. 68, I do not compromise on social activities. 79, I have regular contact with my relatives and friends. 75, I never miss out on any weekend shopping or outings with my family. 88, I enjoy my work. 69, The workplace is good and friendly. 76, I enjoy working in my company. 87, Work makes me creative. 85. Here, all the loadings have an incredible value, confirming the model is a fit.

**Table 11:** CFA factor loadings on family, personal life, and workplace enjoyment.

**Statements**	**Label**	**Statements**	**Factor loading**
Work and family	A1	My work does not interfere with my family's activities	0.75
	A2	I do not feel materially tired when I come home from work	0.73
	A3	My efforts do not affect the amount of time spent on my personal	0.77
	A4	I have adequate time to interact with family members due to work overload	0.70
Personal life	A5	I have time for my activities	0.68
	A6	I do not compromise on social activities	0.79
	A7	I have regular contact with my relatives and friends.	0.75
	A8	I never miss out on any weekend shopping or outings with my family	0.88
Workplace enjoyment	A9	I enjoy my work	0.69
	A10	The workplace is good and friendly	0.76
	A11	I enjoy working in my company	0.87
	A12	Work makes me creative	0.85

## 7. Discussion

The estimation apparatus, created on this basis in light of the proposed build, integrates both theory and practice. Due to its content legitimacy, internal consistency dependability, and confirmations of both united and discriminant legitimacy, the developed scale provided a psychometrically stable estimation of employee commitment. This tool will contribute to the dedication speculation because it was supposed to gauge employee engagement and now no longer measures dedication (Saks, 2006; Rich et al., 2010), authoritative dedication (Saks, 2006), views dedication (Schaufeli et al., 2002) or scholarly, social or emotional dedication (Soane et al., 2012). The assessment tools created and offered within the context of this study are explicitly based on a hypothetical structure that relies on unloading the entire range of employee engagement expertise. Several experts have also added those gadgets due to overt repetition and a lack of genuine content (Macey and Schneider, 2008; Cole et al., 2012; Saks and Gruman, 2014; Nimon et al., 2016). The device created by Pati (2012) in the Indian industry emphasized employee loyalty and promoted content legitimacy. This tool takes into consideration operational errand execution and its aspects, which might be referred to as the conduct effects of employee dedication (Salanova et al., 2005; Rich et al., 2010; Christian et al., 2011; Soane et al., 2012). Taking a look at the shortage of assessment tools in the Indian industry. Designed to remedy the challenges associated with social contrasts in Western evaluation technologies, the scale made a significant commitment to the commitment hypothesis, paying special attention to the Indian context. The estimation tool will serve as a measuring stick for dedication degrees in Indian institutions. HR heads, scholars, and others gave a comprehensive view of consultant dedication. They embodied the atmosphere of employee dedication in many of India's leading commercial enterprise institutions.

## 8. Limitations

All research works are subject to various limitations and this study is not an exception. One of the limitations of this study is the exclusive use of psychometric tests to measure engagement. Although the psychometric tests used in the study are reliable and valid, there may be other legitimate tools available to evaluate employee engagement that were not used. Future research could benefit from the exploration of additional methods to measure employee engagement, such as those that assess aspects of intellectual growth, emotional responsibility, and job fit.

This study confirms the usefulness of psychometric tests in various contexts and organizations. For instance, 385 female IT employees working in three large IT parks in Kerala participated in this research. As a result, participants were not required to disclose personal information such as age, orientation, experience, salary level, and specific socio-economic characteristics, which safeguarded participant confidentiality. Consultants can help researchers identify, establish, and regulate cultural, socioeconomic, and viewpoint groups to determine the generalizability of this methodology in diverse settings.

## 9. Conclusion

The issue of work-life balance has a direct impact on the performance of an organization and its employees. Work-life balance involves controlling when and how people perceive it. Employees maintain a balance between vision and character by utilizing a number of variables as support. Contributing factors, such as employees' cooperation in defining procedures, making critical choices, and effectively complying with institutional techniques can help adjust employees' views and character. Each individual spends the majority of their energy on near images (Chebat et al., 2006). This is why it is critical to maintaining a balance so they can enjoy their spare time with their loved ones. It is evident from the material presented that the vast majority of the research has focused on a few theories, specifically the Spillover Hypothesis, the Conflict Hypothesis, the Segmentation-Integration Hypothesis, the Enrichment-Facilitation Hypothesis, and the Borderline Hypothesis, while the other previously mentioned hypotheses are a unique case in research. This is why the previous speculations were largely based on the balance between fun and serious activities. In the meantime, the hypotheses in the last classification were being considered within the sphere of different sociologies, such as human sciences, brain research, and framework theories; in recent years, momentum research has shifted from negative and clashing perspectives to a more constructive approach. Despite the assumptions presented, the writing can be interpreted as illustrating a few different approaches, such as Human Capital Theory, Social Identity Theory, and Role Theory, among others. This examination of these speculations is primarily humanistically driven and dated; therefore, they have not been discussed above. The hypotheses examined in the paper were discussed in a fundamentally unrelated manner. However, there are usually areas of convergence between these speculations, and it tends to be difficult to distinguish them from one another. The paper has attempted to show differences between beliefs in terms of focus and origin. The level of performance of female employees is compared with the acceptance of telework; ANOVA is critical, so relapse is considered. As the analysis shows, assuming that WFH affects the compliance of specialists increases compliance by 33%, which is a good sign. Invalid speculation is disregarded, recognizing the importance of evidence. We also observed that employees are very satisfied with their organization's psychometric assessments.

## Data availability statement

The raw data supporting the conclusions of this article will be made available by the authors, without undue reservation.

## Ethics statement

Ethical approval was not required for the study involving human participants in accordance with the local legislation and institutional requirements. Written informed consent to participate in this study was not required from the participants in accordance with the national legislation and the institutional requirements.

## Author contributions

Material preparation, data collection, and analysis were performed by BS, AL, GR, and NK. The first draft of the manuscript was written by BS and all authors commented on previous versions of the manuscript. All authors read and approved the final manuscript. All authors contributed to the study's conception and design.
