# Corrigendum: Emerging work environments in the pandemic era: a gendered approach to work-life balance programs

**DOI:** 10.3389/fsoc.2023.1219220

**Published:** 2023-05-30

**Authors:** B. Sreya, Ayyagari Lakshmana Rao, G. Ramakrishnan, Nikhil Kulshretha

**Affiliations:** ^1^SRM University, Amaravathi, India; ^2^Doon Business School, Dehradun, India

**Keywords:** working from home, work-life balance, psychometric testing, satisfaction level of employees, COVID-19 pandemic

In the published article, there was an error in affiliation 2. The affiliation was incorrectly written as “The Doon School, Dehradun, India,” it should be “Doon Business School, Dehradun, India.”

The authors apologize for this error and state that this does not change the scientific conclusions of the article in any way. The original article has been updated

